# Red cell distribution width as a prognostic indicator in medical and surgical ICU patients

**DOI:** 10.3389/fmed.2025.1671323

**Published:** 2025-09-17

**Authors:** Aski Vural, Fadime Cinar, Semra Bulbuloglu, Arife Karamelek

**Affiliations:** ^1^Department of Internal Medicine, Faculty of Medicine, Adiyaman University, Adiyaman, Türkiye; ^2^Division of Surgical Nursing, Department of Nursing, Faculty of Health Sciences, Istanbul Nisantasi University, Istanbul, Türkiye; ^3^Department of Midwifery, Faculty of Health Sciences, Istanbul Aydin University, Istanbul, Türkiye; ^4^Division of Surgical Nursing, Department of Nursing, Faculty of Health Sciences, Istanbul Aydin University, Istanbul, Türkiye

**Keywords:** biomarker, intensive care unit, mortality, prognosis, red cell distribution width, RDW

## Abstract

**Background:**

Red cell distribution width (RDW) has been investigated as a clinical predictor in different study populations. However, its prognostic usefulness in medical and surgical intensive care units remains unknown. This study investigates the role of RDW as a prognostic factor in this specific patient population.

**Objective:**

This study examined the relationship between red cell distribution width and other blood parameters as a prognostic indicator among patients treated in medical and surgical ICU.

**Methods:**

This study is observational in type and its sample consisted of *n* = 197 patients treated in the medical and surgical ICU (msICU) of a public hospital of Istanbul and during 2023. In this study, personal characteristics (age, gender etc.), clinical characteristics (comorbidity, presence of catheters, length of stay etc.) and clinical/laboratory blood parameters (the RDW coefficient of variation (CV), C reactive protein (CRP), albumin, platelet (PLT) etc.) of msICU patients were analyzed. The relationship between clinical/laboratory parameters was examined using the Pearson correlation test, and changes in RDW values according to patient characteristics were examined using ANOVA and independent sample *t*-tests.

**Results:**

In this study a positive, strong and statistically significant relationship existed between RDW and lactate (*r* = 0.704, *p* = 0.004) and CRP (*r* = 0.759, *p* = 0.026), and creatinine (*r* = 0.729, *p* = 0.001). It was reported that a negative, weak and statistically significant relationship existed between RDW and albumin (*r* = −0.172, *p* = 0.015) and PLT (*p* = −0.169, *p* = 0.011). The patient characteristics such as inotropic and vasopressor use, multiple comorbidities, APACHE II score, and surgical experience were factors that increased RDW levels (*p* < 0.05). Patients in the postoperative period and patients with pressure sores had higher RDW values and these differences were statistically significant (*p* < 0.05).

**Conclusion:**

As a result of this study, RDW level was associated with levels of lactate, CRP, albumin, platelet and creatinine among patients in the medical and surgical ICU. The patients with chronic wounds, comorbidities, and/or certain medications and in deceased patients, were associated with increased RDW. RDW may be a useful marker as a prognostic criterion to validate clinical status.

## Introduction

A decrease in hemoglobin concentration and/or absolute red blood cell count is common in critically ill patients, with an incidence level approaching 69.9% (1). Red cell distribution width (RDW) is measured from complete blood count (CBC) and red blood cell histograms and provides information about the size and distribution (anisocytosis) of red blood cells in the vascular circulation (2). So far, there have been several studies suggesting that RDW may be a biomarker for predicting the prognosis of patients with a wide range of conditions including iron deficiency anemia and thalassemia carriage (3, 4), severe burn injuries (2, 5), cancer (6, 7) and coronary artery disease (8). In a study of 1,379 intensive care unit patients, high RDW levels were associated with death and hospital readmission. However, the adequacy of the blood parameters examined in the aforementioned study is controversial and there was no information on how the care and treatment processes of the patients occurred after they were transferred to the clinic (9).

Any physiological condition triggers the release of reticulocytes into the circulation, resulting in an increase in RDW. RDW elevation has not yet been recognized as a disorder, developing secondary to other erythrocyte tests, and there is still no evidence to date as to whether it is a predictor of significant failure. It is thought that elevated RDW levels may be associated with worsening lung function in adult patients (10). There is no direct treatment protocol for elevated RDW associated with mortality. The standard deviation of erythrocytes is obtained by dividing the red blood cell (RBC) by the mean corpuscular volume (MCV) and provides data on erythropoiesis (11).

Mortality of patients hospitalized in the intensive care unit has been associated with several factors. These factors include hypoalbuminemia (12), acute renal failure (13), respiratory (14) and cardiac (15) failure. The problems that lead to the death of patients in the intensive care unit (ICU) who receive medical and surgical treatment may occur simultaneously and initiate a multidimensional process. When this mostly progressive process involves irreversible changes, death may be inevitable. Similarly, a disorder that has started in the ICU or a comorbid disease with aggravated symptoms may be a trigger for another failure (16–18). Previous studies have reported that RDW, in addition to being a chronic inflammatory marker, is associated with sepsis and poor clinical outcomes in the general ICU population (19–21). Meynaar et al. reported that 387 of 2,915 patients (13.3%) treated in the ICU died. The same study highlighted an increase in RDW as an independent predictor of mortality (OR for each fL SD-RDW increase, 1.04; 95% CI: 1.02–1.06) (22). An increase in RDW may be associated with prognosis in patients who remain in the medical and surgical ICU in relation to or independent of known predictors (albumin, C reactive protein, interleukin-6, etc.). In this study, we examined the relationship between red cell distribution width and other clinical/laboratory parameters (C reactive protein, albumin, platelet, etc.) as a prognostic indicator among patients treated in medical and surgical ICU.

## Materials and methods

This study followed recommendations from STROBE (Strengthening the Reporting of Observational studies in Epidemiology) (23). The type of this study was observational. The data of this study were retrospectively obtained from electronic data and patient files of a medical and surgical ICU (msICU). The data of the patients were recorded in an Excel-based data recording form developed by the researchers. G-power analysis (calculated with the G*power 3.1.9.1 program) was used to calculate the sample size. Considering a margin of error of 0.05 and a 95% confidence interval, at least *n* = 120 patients should have been included in the sample. The sample of this study consisted of *n* = 197 patients. In this study, patients who met the inclusion criteria were included in the sample without using a sampling method. The inclusion and exclusion criteria for the sample of this study are presented below. The inclusion criteria for this study are as follows: (i) being treated in the msICU of the hospital where the study was conducted in the year 2023, (ii) having an ICU stay of at least 10 days, (iii) being 18 years of age or older constituted the inclusion criteria. In contrast to the inclusion criteria, patients with missing data records were excluded. Data for this study were collected retrospectively in the msICU of a public hospital. Retrospectively, data were extracted from patient files and electronic records, and the obtained data were recorded by researchers on excel sheets with unidentified patients. Patients treated in the msICU of a public hospital in western Turkey during between January 1, and December 31, 2023 constituted the sample. In this study, independent variables were included time of ICU stay (Days), time to intubation (Days), duration of central venous catheter (CVC) and percutaneous endoscopic gastrostomy (PEG), of foley stay and of antibiotic use, number of blood product transfusions, duration of inotropic and vasopressor use, gender, reason for ICU admission and type, neurological disorders, presence of chronic disease, presence of pressure wound, feeding catheter, nosocomial infection. In addition, CCI and APACHE II scores were also independent variables. The outcome variables in this study are organ (liver, lung, heart and kidney) function tests, coagulation factors, immune function tests, infection panel and erythrocyte tests.

### Data collection tools

Intensive care unit clinical characteristics form, Charlson comorbidity index, and Acute Physiology And Chronic Health Evaluation (APACHE) II scoring (scoring is performed at the initial hospitalization) were used for data collection. Information about the data collection tools is given below.

### Intensive care clinical characteristics form

The data recording form included questions about the age, gender, clinical characteristics, information on probes and catheters, the drug information, infections and disorders, organ function tests, blood parameters, etc.

### Charlson comorbidity index

The Charlson comorbidity index (CCI) was developed by Charlson (24). When the CCI is examined, it is seen that the condition of the patients and a total of 19 morbidities are addressed. A scoring is created according to the presence of these conditions and morbidities. A 1-year mortality prediction is made using CCI. In this context, many comorbid diseases and conditions are categorized. Calculations are made according to the risk level and the scoring is between 1, 2, 3 and 6. Mortality is predicted by summing the scores.

### APACHE II scoring

Acute Physiology And Chronic Health Evaluation (APACHE) II scoring, a predictor scoring system for mortality, was developed by Knaus et al., in 1985 and has been used in mortality prediction since then (25). APACHE II predicts mortality by taking into account age, gender, blood pressure, heart and respiratory rate, partial oxygen pressure and oxygen requirement of the patient, and other blood parameters.

## Statistical analysis

The retrospective data were coded into the Statistical Package for Social Sciences (SPSS) for IBM 27. Normal distribution assumptions were calculated by Kolmogorov-Smirnov analysis (*p* > 0.05). Statistical results were evaluated with 95% confidence interval and *p* < 0.05 significance level. Age, gender, clinical characteristics, kateterlerin kalış gün sayısı, organ function tests, blood parameters, CCI ve APACHE II skorları were used to calculate with frequency (%) veya descriptive tests [Standard Deviation (Sd), Mean, Minimum (Min.) and Maximum (Max) values]. The relationship between categorical variables (gender, ICU type, presence of ulcer, etc.) and continuous variables (blood values) was evaluated by independent sample *t*-test, ANOVA one-way analysis of variance, regression analysis The relationship between two continuous variables (albumin, lactate, CRP, PLT, Creatinine, and Hemoglobin) was determined by Pearson correlation test.

### Ethical aspects of the study

The data of this study were approved by the Institutional Review Board of Esenyurt Necmi Kadioglu State Hospital, Department of Anesthesia and Reanimation before being extracted from electronic and written patient records. Ethics Committee Permission was obtained from Istanbul Aydın University Clinical Research Ethics Committee (Date: 31.07.2024, Number: 71/2024). The research steps were followed by the Declaration of Helsinki, patient confidentiality was respected, and no private and personal data of the patients were accessed.

## Results

Table 1 shows the characteristics of patients and mean RDW values together with parametric test results. The mean age of the intensive care unit patients in this study was 70.51 ± 16.86 years, CCI score was 2.51 ± 1.38, and the APACHE II score was 26.37 ± 7.44. Inotropic and vasopressor use was 7.27 ± 8.10 per day. Deceased patients in 59.4% of intensive care unit patients. There was a positive, weak and statistically significant relationship between the duration of inotropic and vasopressor use and RDW level (*r* = 0.185, *p* = 0.009). There was a positive, moderate and statistically significant relationship between the APACHE II score (*r* = 0.465, *p* = 0.020) and CCI score (*r* = 0.342, *p* < 0.001) and RDW level. Those with surgical history and pressure sores had higher RDW values and these differences were statistically significant (*p* < 0.05). Deceased patients had higher RDW values and this difference was statistically significant (*p* < 0.01).

**TABLE 1:** Characteristics and mean RDW values of patients in msICU.

Characteristics	Mean ± Sd (Min., Max)		RDW-CV	
			Mean ± Sd	Test and Sig.
Age	70.51 ± 16.86 (20, 99)		17.10 ± 3.04	*r* = 0.001, *p* = 0.989
Time of ICU stay (days)	25.13 ± 17.31 (10, 84)			*r* = 0.086, *p* = 0.728
Time to intubation (days)	16.41 ± 10.52 (0, 77)			*r* = 0.114, *p* = 0.111
Duration of CVC stay (days)	12.22 ± 9.34 (0, 74)			*r* = 0.112, *p* = 0.117
Duration of foley stay (days)	24.80 ± 17.6 (0, 84)			*r* = 0.097, *p* = 0.175
Duration of antibiotic use (days)	29.75 ± 21.12 (0, 127)			T
Number of blood product transfusions	7.21 ± 3.17 (0, 17)			*r* = 0.102, *p* = 0.155
Duration of inotropic and vasopressor use (days)	7.27 ± 8.10 (0, 41)			*r* = 0.185, *p* = 0.009*
Duration of LMWH (days)	12.34 ± 4.66 (0, 27)			*r* = 0.321, *p* = 0.446
CCI score	2.51 ± 1.38 (0, 5)			*r* = 0.342, *p* < 0.001**
APACHE II score	26.37 ± 7.44 (10, 44)			*r* = 0.465, *p* = 0.020*
	***n***	**%**		
**Gender**				
Woman	110	55.8	17.16 ± 2.90	*t* = 0.328, *p* = 0.904
Male	87	44.2	17.02 ± 3.22	
**Reason for ICU admission**				
Acute respiratory disorders	157	79.7	16.93 ± 3.04	*F* = 1.258, *p* = 0.274
Sepsis	14	7.1	18.07 ± 2.45	
Neurological disorders	13	6.6	17.13 ± 3.14	
Advanced stage (4 and 5) malignancy	4	2	21.36 ± 5.39	
Other causes (cardiac, renal, traumatic)	9	4.6	17.10 ± 2.99	
**Presence of chronic disease**				
Yes	188	95.4	17.13 ± 3.07	*t* = 0.740, *p* = 0.460
No	9	4.6	16.36 ± 2.40	
**ICU type**				
Surgery	60	30.5	17.91 ± 3.66	*t* = 2.522, *p* = 0.012*
Medical	137	69.5	16.74 ± 2.67	
**Presence of pressure wound**				
Yes	90	45.7	17.62 ± 2.79	*t* = 2.227, *p* = 0.027*
No	107	54.3	16.66 ± 3.18	
**Feeding catheter**				
PEG	30	15.2	17.14 ± 1.38	*t* = 0.791, *p* = 0.533
NG catheter	126	64	17.08 ± 3.68	
Peroral	41	20.8	17.11 ± 2.14	
**Nosocomial infection**				
Yes	145	73.6	17.06 ± 2.58	*t* = 0.076, *p* = 0.784
No	52	26.4	17.20 ± 4.09	
**Conclusion**				
Death	117	59.4	17.74 ± 2.83	*t* = 1.437, *p* < 0.001**
Transfer to clinic	80	40.6	16.16 ± 3.11	

Statements with statistical significance are shown in bold.

*p < 0.05,

**p < 0.01; *r*, Pearson correlation coefficient; *F*, Oneway ANOVA, *t*, Independent sample *t*-test. RDW-CV, Red Cell Distribution Width Coefficient of Variation; LMWH, Low Molecular Weight Heparin.

Table 2 shows the values of coagulation factors, immune function and erythrocyte test values of patients msICU. When the values of coagulation factors of intensive care unit patients were examined, it was determined that the mean Platelet (PLT) and Activated Partial Thromboplastin Time (APTT) were within the normal reference range. International Normalized Ratio (INR) and Fibrinogen levels were elevated. D-dimer level had a very high mean value almost 5 times above the maximum value (2747.20 ± 1013.14). Neutrophil (79.22 ± 13.86), White Blood Cell (WBC) (14.05 ± 11.14), CRP (145.76 ± 125.91) and procalcitonin (3.35 ± 5.29) levels were well above the average when immune function tests and infection panel values of intensive care unit patients were analyzed. When the values of erythrocyte tests of intensive care unit patients were analyzed, the mean RDW (RDW-CV: 17.10 ± 3.04) was above the reference value, similarly, the mean ferritin (404.76 ± 193.25) was well above the reference value. Hemoglobin (HGB) (9.17 ± 1.90), Hematocrit (HCT) (29.23 ± 6.14), Red Blood Cell (RBC) (3.23 ± 0.75) and Mean Corpuscular Haemoglobin Concentration (MCHC) (28.56 ± 2.57) were below the reference values.

**TABLE 2:** Coagulation factors, immune function and erythrocyte test values.

Blood parameters	Mean ± Sd	Min. – Max.
**Coagulation factors**
INR (0.8 – 1.2)	1.50 ± 1.77	9.96 – 7.75
PLT (150 – 400 10∧9/L)	208.10 ± 45.46	9 – 492
APTT (25 – 39 Sec.)	34.48 ± 20.13	7.50 – 247
Fibrinogen (150 – 450 mg/dL)	553.50 ± 195.57	52.90 – 1,200
D-dimer (<500 ng/mL)	2747.20 ± 1013.14	0 – 35,200
**Immune function tests**
WBC (3.8 – 10 10^3^/μL)	14.05 ± 11.14	0.04 – 64.77
Neutrophil (1.56– 6.13 10^3^/μL)	79.22 ± 13.86	19.70 – 97.20
**Infection panel**
CRP (<5 mg/L)	145.76 ± 25.91	0.60 – 192
Procalcitonin (0–0.05 ng/mL)	3.35 ± 5.29	0.00 – 19.7
**Erythrocyte tests**
RDW-CV (11.2 – 15%)	17.10 ± 3.04	12 – 31.60
HGB (11.5 – 15.5 g/dL)	9.17 ± 1.90	5 – 17.40
Ferritin (20 – 300 μg/L)	404.76 ± 193.25	0 – 821
HCT (35.5 – 48%)	29.23 ± 6.14	14 – 52.20
RBC (3.8 – 5.6 10(6)/μL)	3.23 ± 0.75	0.34 – 6.16
MCHC (31.0 – 37.0 g/dL)	28.56 ± 2.57	18.60 – 36.80
MCV (80 – 95 fL)	90.29 ± 8.66	32.80 – 111

Table 3 shows the values related to organ function tests of patients in msICU. Albumin (23.14 ± 10.04) was well below the reference range, and total protein (62.74 ± 34.06) and alanine aminotransferase (ALT) (41.31 ± 18.16) were within the range. The mean values of aspartate transaminase (AST) (105.69 ± 46.24) and lactate dehydrogenase (LDH) (492.86 ± 196.11) were higher than maximum values.

**TABLE 3:** Organ (liver, lung, heart and kidney) function tests.

Blood parameters	Mean ± Sd	Min. – Max.
**Liver**
Albumin (35 – 54 g/L)	23.14 ± 10.04	0 – 41.60
Total protein (60 – 83 g/L)	62.74 ± 34.06	25.2 – 56.6
AST (8 – 33 U/L)	105.69 ± 46.24	5 – 5.152
ALT (<41 U/L)	41.31 ± 18.16	0.76 – 1.070
LDH (125 – 220 U/L)	492.86 ± 196.11	36.60 – 3.798
Glucose (74 – 106 mg/dL)	146.44 ± 82.17	6 – 545
**Lung**
Lactate (1 – 1.5 mmol/L)	4.96 ± 3.75	0.30 – 26
**Heart**
Troponin T (0 – 0.04 ng/ml)	0.10 ± 0.32	0 – 3.58
**Kidney**
e-GFR (≥90)	79.86 ± 23.88	7 – 115
Urea (7 – 20 mg/dL)	76.34 ± 9.11	8 – 99
Creatinine (0.5 – 1.13 mg/dL)	1.13 ± 1.07	0.01 – 6.50

Table 4 shows the correlation analysis between RDW and Albumin, Lactate, CRP, PLT, Creatinine and Hemoglobin. There was a negative, weak and statistically significant correlation between RDW and albumin (*r* = −0.172, *p* = 0.015). There was a negative, weak and statistically significant relationship between RDW and PLT (*r* = −0.169, *p* = 0.015). There was a positive, strong and statistically significant relationship between RDW and lactate (*r* = 0.704, *p* = 0.004). There was a positive, strong and statistically significant correlation between RDW and CRP (*r* = 0.759, *p* = 0.026). There was a positive, strong and statistically significant correlation between RDW and creatinine (*r* = 0.729, *p* = 0.001). There was a negative, weak and statistically significant correlation between RDW and hemoglobin (*r* = −0.184, *p* = 0.011).

**TABLE 4:** Correlation analysis between RDW-CV and albumin, lactate, CRP, PLT, creatinine and hemoglobin.

		RDW-CV	
Correlation	Blood parameters	*R*	*P*
Pearson correlation	Albumin	−0.172	0.015*
	Lactate	0.704	0.004**
	CRP	0.759	0.026*
	PLT	−0.169	0.015**
	Creatinine	0.229	0.001**
	Hemoglobin	−0.184	0.011*

*r*, Pearson Correlation;

*p < 0.05,

**p < 0.01.

**FIGURE 1:**
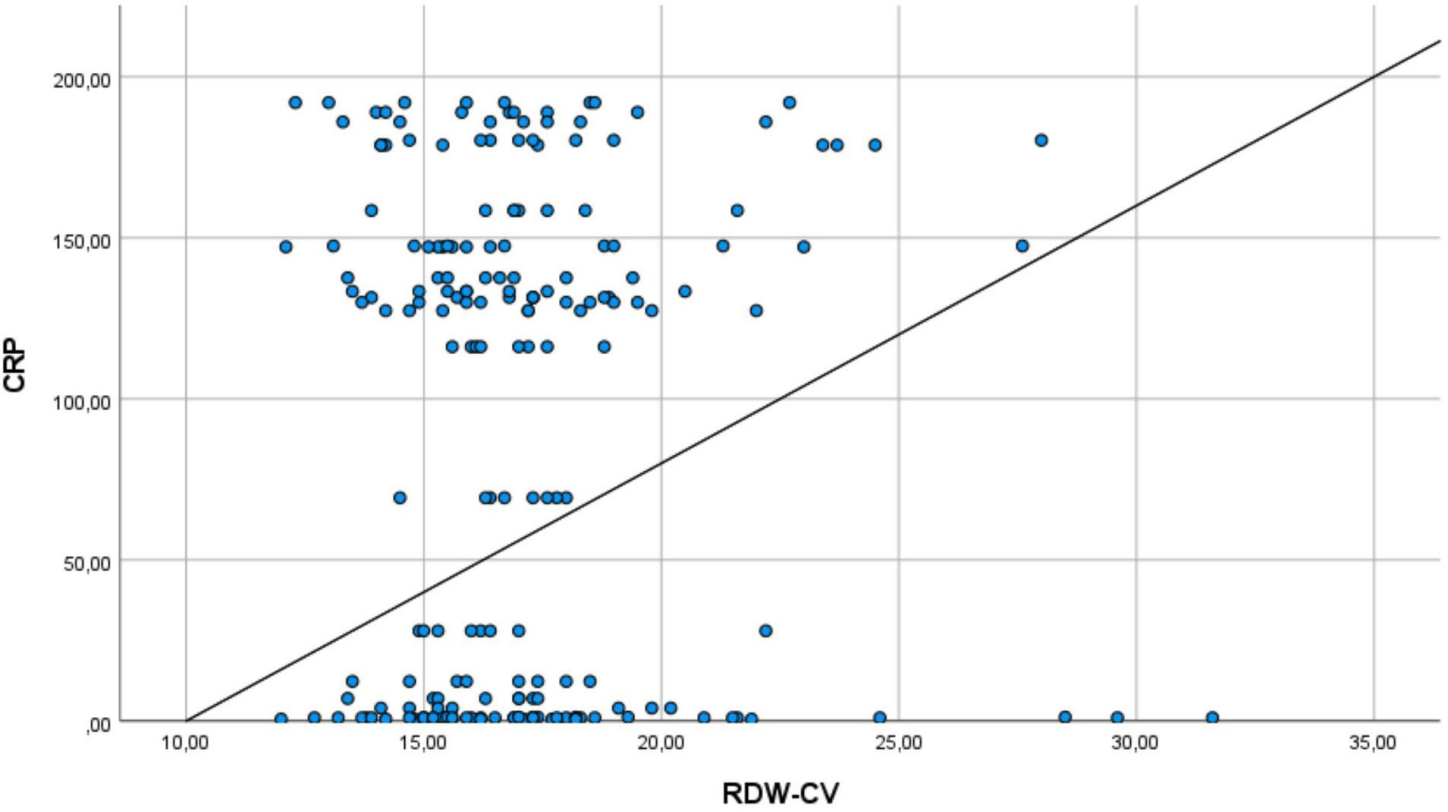
The relationship between RDW and CRP of patients. It shows the same directional relationship between RDW and CRP values in intensive care unit patients.

Figure 1 shows the relationship between RDW and CRP of patients in msICU. There is the same directional relationship between RDW and CRP among patients.

**FIGURE 2:**
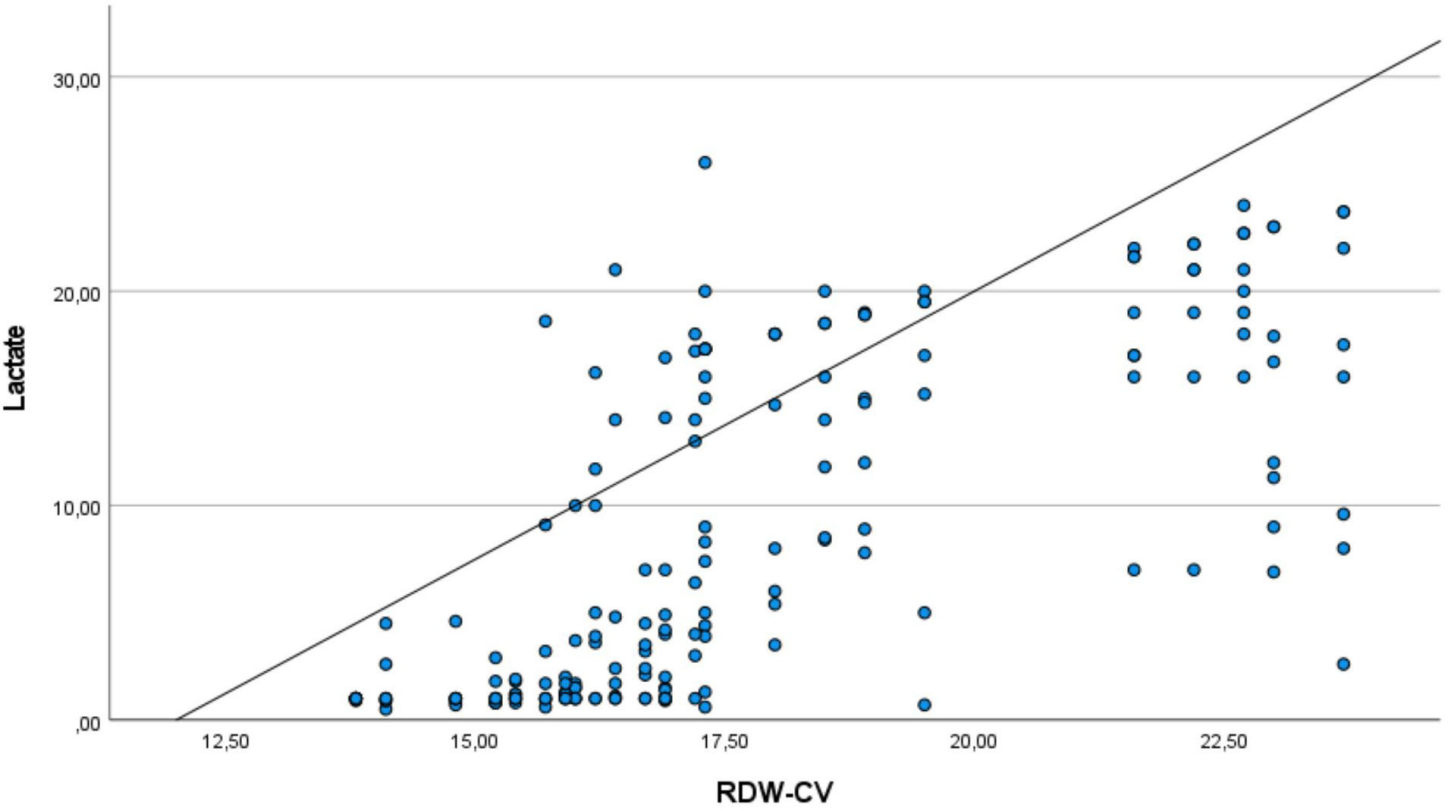
The relationship between RDW and lactate of patients. It shows the same directional relationship between RDW and lactate values in intensive care unit patients.

Figure 2 shows the relationship between RDW and lactate of patients in msICU. There is the same directional relationship between RDW and lactate among patients.

**FIGURE 3:**
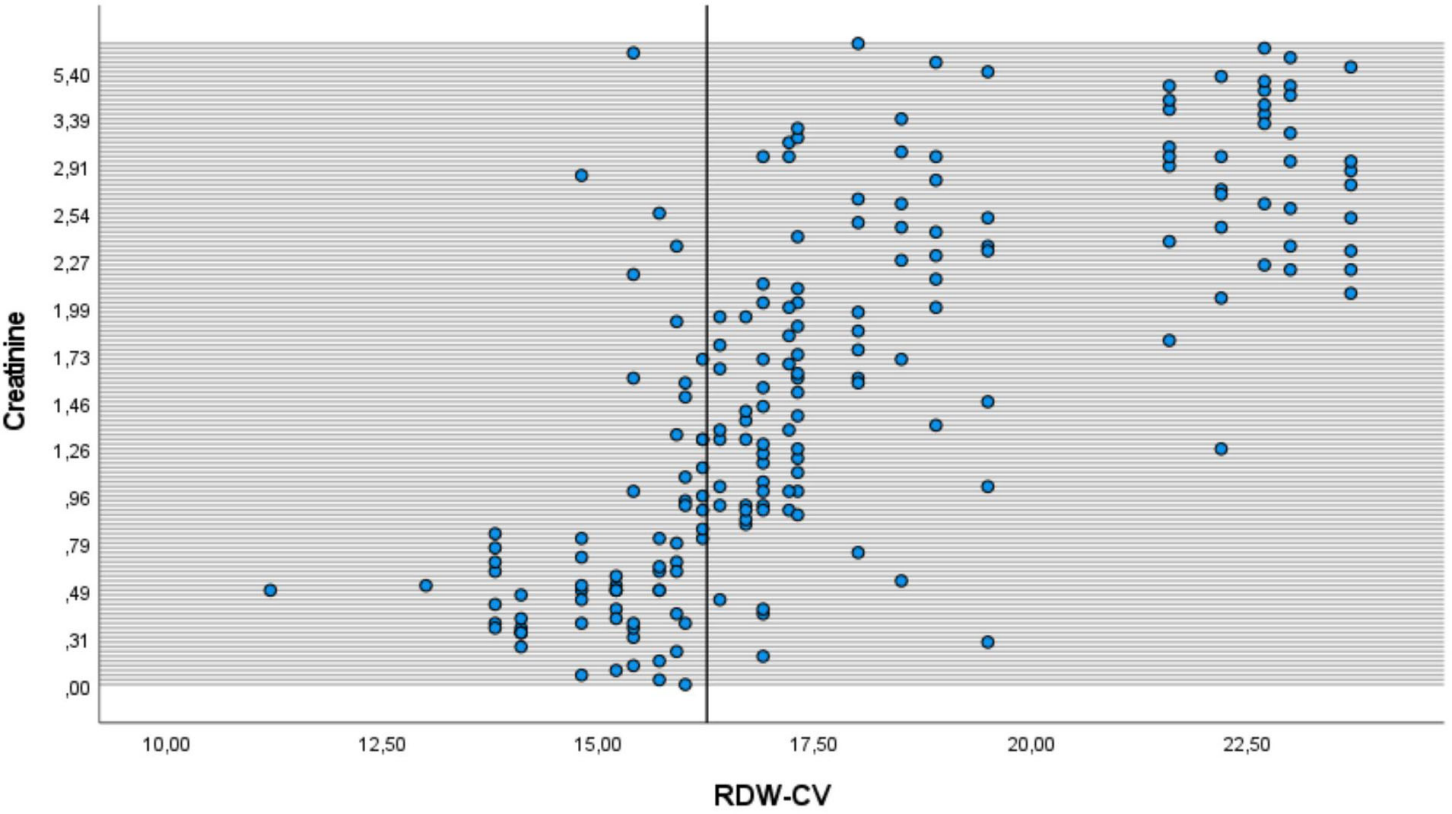
The relationship between RDW and creatinine of patients. It shows the same directional relationship between RDW and creatinine values of intensive care unit patients.

Figure 3 shows the relationship between RDW and creatinine of patients in msICU. There is the same directional relationship between RDW and creatinine among patients.

According to regression analysis, the relationship between death and RDW, and the predictors of RDW-CV are shown in Table 5. RDW-CV is a predictor of death and has a significant effect of 7% (*p* < 0.001). APACHE II, CCI score, and duration of inotropic and vasopressor use were determined to be predictors of RDW-CV and had a significant effect of 21% (*p* < 0.001). Surgery type and the presence of a pressure wound affect RDW-CV by 11% (*p* = 0.012, *p* = 0.027).

**TABLE 5:** The relationship between death and RDW-CV, and the predictors of RDW-CV according to regression analysis, (*n* = 197).

			Unstandardized Coefficients		Standardized Coefficients	Items				
		Model	B	Std. Error	Beta	*t*	*P*	*F*	Sig.	*R* ^2^
Death	1	(Constant)	30.601	1.311		3.3760	.000	10.399	0.001^a^	0.071
		RDW-CV	2.422	0.781	0.318	1.4370	.001			
RDW-CV	1	(Constant)	8.528	1.254		6.8820	.000	5.301	0.001^b^	0.212
		APACHE II	0.371	0.291	0.266	3.4140	.022			
		CCI Score	−0.041	0.044	−0.208	−1.2860	.004			
		Duration of inotropic and vasopressor use	0.721	0.254	0.244	1.7040	.006			
	2	(Constant)	9.021	1.123		8.0300	.000	6.553	0.000^c^	0.111
		ICU Type	0.325	0.119	0.354	2.5220	.012			
		Presence of pressure wound	0.046	0.034	0.310	2.2270	.027			

^a^Predictors: (Constant), Death.

^b^Predictors: (Constant), APACHE II, CCI.

^c^Predictors: (Constant), Surgery Type, Presence of pressure wound.

## Discussion

In this study, we examined the relationship between clinical characteristics, other blood parameters and RDW values of patients treated in the msICU. The RDW level of intensive care unit patients has been associated with mortality in several previous studies. Otero et al. examined the relationship between RDW measured at the first admission to the intensive care unit and mortality, and it was noted that surgical intensive care unit patients with high RDW had an increased risk of 90-day mortality (26). In the same study, the presence of systemic inflammation accompanying the disease requiring intensive care was reported, but no results regarding blood parameters other than RDW were included. Han et al. reported that RDW was a reliable biomarker in predicting 4-year mortality (27). In the study of Nan et al., ventilator-associated pneumonia and mean RDW values were compared with in-hospital mortality rate. As a result of the aforementioned study, it was noted that those who died due to ventilator-associated pneumonia had high RDW levels (28). Similar to the literature, in this study, 59.4% of patients died in the msICU. Those who died had higher RDW levels than those transferred from the msICU. APACHE II, CCI score, and duration of inotropic and vasopressor use were determined to be predictors of RDW-CV. Presence of a pressure ulcer, multiple comorbidities and a high APACHE II score, and prolonged inotrope and vasopressor use were factors that increased RDW levels in this study.

The use of RDW in predicting mortality in serious diseases is undoubtedly due to its variation in the same or opposite direction from previously defined predictors. In the study by Nan et al., it was noted that changing RDW values had no statistically significant effect on lactate (mmol/L) (28). In the literature, it has been reported that blood values associated with RDW are WBC, creatinine, PLT and INR, and RDW increases as the CCI score increases. In our study, INR and PLT levels may have been affected by low molecular weight heparin treatment administered in msICU, and the mean WBC was well above the reference value (14.05 ± 11.14). Our results support the same directional and strong correlation between creatinine and RDW, similar to the study of Nan et al. In addition, RDW-CV explained 7% of the mortality.

The results of our study report the same directional and strong correlation between RDW and lactate in intensive care unit patients, unlike the study of Nan et al. Aras and Paşalı Kilit reported that elevated lactate and RDW were associated with mortality in tertiary intensive care unit patients (29). Bakker et al. reported that lactate measurement was a predictor for multiple organ dysfunction syndrome (MODS) and mortality in patients with septic shock (30). Hyperlactataemia is defined as an increased lactate level and occurs with water, ATP and (lactate)- production as a result of hypoxia when inadequate tissue perfusion develops (31, 32). In this study, the mechanism of the relationship between RDW mean value and lactate mean value could not be determined.

The relationship between increased RDW levels and oxidative stress and inflammatory response has been previously described in intensive care unit patients and is associated with multiple organ failure (20, 33). In this study, the mean CRP level was well above the reference range, but there was a positive, strong and statistically significant correlation between RDW and CRP. A similar strong correlation was found between RDW and creatinine. Elevated creatinine is associated with renal failure (34, 35). MODS is one of the most common causes of death in ICU (36) and can be defined as acute failure of two or more organs (37). Improvement of dysregulated immune response and amelioration of organ damage with various therapeutic options are important components of the treatment protocol (38).

In this study, 7.1% of the patients were hospitalized in ICU due to sepsis, 79.7% due to acute respiratory disorders, 95.4% had a chronic disease, CCI score was 2.51 ± 1.38, and the mean age was 70.51 ± 16.86 years. There was a statistically significant correlation between the duration of inotropic and vasopressor use, CCI score, APACHE II score and RDW levels. In addition, the RDW level of patients who were deceased was higher than those who were transferred to the clinic and this difference was statistically significant. Previous studies have pointed out that there may be a link between renal failure, oxidative stress, cardiac problems, increased neuroendocrine activities and hypoalbuminemia and elevated RDW (2, 39–41).

In the literature, there is an opinion that albumin infusion does not reduce mortality even if it reduces hypalbuminemia (42). Another study argued that there was no relationship between hypoalbuminemia and mortality and associated MODS with low albumin (43). In our study, the correlation between albumin and RDW was negative and weak; however, the mean albumin value was found to be below what it should be. In this study, decreased hemoglobin levels were associated with increased RDW. This correlation was weak and statistically significant. Previous a study by Wubet et al., reported that anemia (hemoglobin level of less than average 12 g/dl) was seen in two-thirds of patients in the surgical ICU (1). In another study by Wubet et al., were reported correlations between anemia and polypharmacy, multimorbidity, and a history of major surgery. In our study, anemia increased RDW levels, and patients with higher RDW had multiple comorbidities. Furthermore, surgical patients had higher RDW levels than those receiving medical therapy (44). Various studies in the literature partially support our results. In addition, the fact that we addressed many patient characteristics and blood values in the same framework with RDW reveals the strength of this study.

This study has several limitation principles. This study had a retrospective single-center design, and the number of patients with common ICU comorbidities was in the minority. There was no information regarding the calibration and bias of the measuring instruments used in the laboratory. In addition, small sample sizetherapeutic approaches, medications, nutritional products, blood values at initial hospitalization and unknowns regarding the current nutritional status were considered as limitations.

## Conclusion

Considering the clinical characteristics of this study, the relatively high mean age, the presence of multiple comorbidities, a high APACHE II score, the use of inotropic and vasopressor medications, and a long ICU stay suggest that the prognosis is generally poor. The study results suggest that these clinical features can associated with increased RDW levels, although the mechanism is not explained. According to the results obtained in our study, increased RDW was weakly associated with decreased albumin and hemoglobin. In contrast, lactate, CRP and creatinine increased together with RDW. The results obtained in our study can guide clinicians during the management of intensive care unit patients. We recommend examining RDW levels in medical and surgical ICU patients in larger sample sizes and multicenter studies.

## Data Availability

The raw data supporting the conclusions of this article will be made available by the authors, without undue reservation.
